# Supplementary material for: Dispositional and situational personal features and acute post-collision head and neck pain: Double mediation of pain catastrophizing and pain sensitivity
Source: PLoS One. 2022 Jan 10;17(1):e0262076. doi: 10.1371/journal.pone.0262076 (PMC8746745; doi:10.1371/journal.pone.0262076)
Supplement: Attachment — Submitted filename: PLOS ONE Response to Reviewer 23.9.21.docx [file pone.0262076.s003.docx]

PONE-D-20-40180R1
Dispositional and situational personal features and acute post-collision head and neck pain: Double mediation of pain catastrophizing and pain sensitivity
PLOS ONE

Dear Jovana Bjekic and Ljiljana B. Lazarevic,

We would like to thank you for taking the time to review our manuscript entitled “Dispositional and situational personal features and acute post-collision head and neck pain: Double mediation of pain catastrophizing and pain sensitivity”. We have made the minor changes that were requested and hope that the editor and reviewers will be satisfied with this version of the manuscript. Please find our responses below.

Sincerely yours,

The authors

*Editorial comments:

Please review your reference list to ensure that it is complete and correct. If you have cited papers that have been retracted, please include the rationale for doing so in the manuscript text, or remove these references and replace them with relevant current references. Any changes to the reference list should be mentioned in the rebuttal letter that accompanies your revised manuscript. If you need to cite a retracted article, indicate the article’s retracted status in the References list and also include a citation and full reference for the retraction notice.*

**Our response:** Thank you for pointing this out. We have updated many recent citations and removed some of the older ones during the previous revision. This process resulted in an error in the reference list. Based on this comment, we double-checked all citations and references and made sure they were correct and complete.

*Reviewer #1*

*I believe authors have addressed all the major concerns in a reasonable manner; I like that they reanalyzed the data and added new participants. The intro and discussion are much better written now.*

**Our response:** We appreciate your positive feedback. We have indeed made a major revision of the manuscript, and we are pleased that you are satisfied with it.

*Reviewer #2*

*Thank you for making changes in the manuscript. I identified some minor issues that should be fixed:
1. Check reference mentioned in line 58 – reference number 6 is missing.*

**Our response:**  Thank you for pointing this out. During the revision process, reference #6 was accidentally omitted from the text. It has now been added as reference #2. The text and reference list have been corrected accordingly.

*2. Openness to changes should be openness to experiences – please revise it in whole manuscript*

**Our response:**  Thank you for this comment. We used the term open to experiences rather than Open to changes throughout the manuscript, including tables and figures.

*3. Encore – wrong spelling, it should anchors.*

**Our response:**  In line with this comment, the typo was corrected.

*4. Correlations should be presented with 2 decimals.*

**Our response:**  We have corrected both the correlation table and the correlation information in the text to show 2 decimals.
